# Awareness of insomnia caused by coffee consumption among adults in Jazan, Saudi Arabia

**DOI:** 10.1097/MD.0000000000039784

**Published:** 2024-09-20

**Authors:** Rama M. Chandika, Fatima A. Elfaki, Abdulrahman A. Alsayegh, Husameldin E. Khalafalla, Emadaldeen A. Alsayed, Hussin M. Hadi, Nasser M. Alhazmy, Fahed A. Aqili, Ahmed H. Al-Hadi

**Affiliations:** a Department of Clinical Nutrition, College of Nursing and Health Sciences, Jazan University, Jazan, Kingdom of Saudi Arabia; b Research Institute of Nutrition and Translational Research in Metabolism, Maastricht University, Maastricht, The Netherlands; c Department of Health Education and Promotion, Maastricht University, Maastricht, The Netherlands.

**Keywords:** Arabic coffee, chi-square test, coffee consumption, insomnia, Saudi Arabia

## Abstract

The Kingdom of Saudi Arabia is the second largest country in the Arabian Peninsula and ranks 61st out of 150 countries in terms of coffee consumption. Excessive coffee consumption is associated with the risk of adverse health outcomes. This study aimed to assess awareness of the effects of coffee consumption and its relationship with insomnia among the adult population in Jazan, Saudi Arabia. A cross-sectional study was conducted with 431 adults in the Jazan region. A self-administered questionnaire was distributed to the participants using an online survey. Data were analyzed using Statistical Program for the Social Sciences, Version 24.0. Of 431 participants, 377 (87.5%) consumed coffee. Females and married women consumed more coffee than their counterparts (91.3%, *P* < .01; 92.8%, *P* < .05; respectively), while students consumed coffee (85.2%) less than both the employed and unemployed (*P* < .05). The proportion of consumption increased from underweight (79.7%) to normal weight (88.5%), and as high as 91.3% among overweight/obese (*P* < .05). More than one-third of the participants (35.5%) preferred drinking coffee at coffee shops. The most preferred coffee was Arabic coffee (40%), and the major reason for drinking coffee was to change their mood (29.7%). More than half of the participants (54.5%) reported that coffee caused insomnia (*P* < .01). Awareness-raising initiatives for the negative effects of excessive coffee consumption are important. Longitudinal studies are required in the next stage of research to identify trends such as the motivations and profiles of Saudi coffee drinkers.

## 1. Introduction

Coffee is one of the most consumed beverages, and caffeine is the most widely used psychoactive substance in the world,^[1]^ and unlike many of them it is legal and widely accessible. Widespread habitual consumption of coffee means that even minor health effects can have significant public health consequences.^[2]^

Coffee is an intricate blend of several biologically active compounds. In addition to caffeine, which functions as both a central nervous system stimulant and bronchodilator, caffeine also contains substances that boast coffee’s antioxidant and anti-inflammatory effects, such as melanoids, quinides, lignans, and trigonelline, as well as chlorogenic acid, which is also recognized for its positive impact on glucose metabolism, and diterpenes, known for their potential to elevate serum cholesterol.^[2,3]^

Caffeine (1,3,7-trimethylxanthine) is responsible for the habit-forming nature of coffee,^[2]^ and has been found to act like some drugs of dependence and produce similar behavioral and physiological effects, as outlined by a review by Meredith et al (2013).^[4]^ The majority of caffeine intake was from coffee consumers 36% then 23% tea and energy drinks 5% respectively.^[5]^

The amount of caffeine in coffee can vary considerably^[2]^ depending on the method of brewing/preparation/type of beans used, size of the serving, and coffee-to-water ratio.^[6,7]^ The permissible dose of caffeine per day is between 300 and 400 mg/day, which equates to approximately 3 to 4 cups times per day.^[6,8]^ There are different types of coffee (e.g., instant, espresso, Turkish), which differ in their caffeine and other ingredients. Arabian coffee, also known as “Qahwa” or “Gahwa,” is traditionally prepared using lightly roasted Arabica coffee beans and is a significant part of Saudi Arabian culture. In amounts equivalent to daily consumption of each type, 1 cup of Turkish coffee (60 mL) contained 20 times the caffeine found in the usual number of cups of Arabian coffee consumed per day (6 cups of 25 mL) and 2 Nescafe® cups (2 cups of 150 mL).^[7]^ The widespread use of coffee- and caffeine-containing foods has been the focus of many studies and reviews that have explored their effects on health. Table 1 summarizes the main findings of these studies. It should be noted that the effect of caffeine is likely to depend on the dose, time of administration/consumption, and the level of caffeine sensitivity and naivety along with beverage type,^[20]^ and that many of the harmful risks of coffee and caffeine consumption are observed more in heavy consumption and/or in coffee-naïve individuals.

**Table 1:** Health effects of coffee/caffeine.

Health condition	Reference	Main conclusion
Health benefits		
Parkinson disease and Alzheimer disease	Wierzejska (2017)^[9]^ Ascherio et al (2001)^[10]^ Quintana et al (2007)^[11]^ Xu et al (2015)^[12]^	Inverse relationship was found in many original studies and reviews between coffee consumption and Parkinson disease and Alzheimer disease. The evidence appears to be weak, however, and dose-dependent (specified as moderate by some studies).
Stroke	Van Dam et al (2020)^[13]^	Many prospective studies and reviews, and meta-analyses have reported a weak inverse association between moderate consumption of coffee and risk of stroke.
Cognitive Performance.	Van Dam et al (2020)^[13]^ Nehlig (2010), Nehlig et al (1992)^[5,14]^	Caffeine reduce fatigue, increase alertness, and reduces reaction time, enhances cognitive functions, and uplifts mood perhaps by its antagonizing effect to adenosine.
Diabetes T2D	O’Keefe et al (2018)^[2]^	In some long-term studies coffee reduced the risk of type 2 diabetes.
Obesity	O’Keefe et al (2018); Sirotkin & Kolesarova (2021)^[2,15]^	Regular coffee consumption may mitigate the potential for obesity in individuals with a genetic predisposition to the condition.
cardiovascular mortality	Poole et al (2017b)^[16]^	The risk of coronary artery disease, and death from cardiovascular causes are found to have an inverse relationship with coffee consumption, with the consumption of 3 to 5 cups per day associated with the lowest risk.
Cancer and liver diseases	Poole et al (2017b)^[16]^	Consumption of coffee is associated with a lower risk of several types of cancer and neurological, metabolic, and liver cancer and cirrhosis associated with coffee consumption.
Depression	Jee et al (2020)^[17]^	Dose-dependent: moderate caffeine intake has a positive effect on depression. , while excessive caffeine intake can exacerbate depression.
All-cause mortality	O’Keefe et al (2018); Poole et al (2017b)^[2,16]^	A dose-dependent reductions (to 400 mg/day) in all-cause mortality in a number of studies.
As a drug: Pain relief	Van Dam et al (2020)^[13]^	Caffeine synergizes with analgesics for better relief of pain.
As a drug: apnea of prematurity	Skouroliakou et al (2009)^[18]^	The use of caffeine might carry an advantage over theophylline for premature infants.
Health risks		
All-cause mortality	Liu et al (2013)^[19]^	Excessive coffee consumption (more than 28 cups/week) has positive association with all-cause mortality in men and in men and women younger than 55 years.
Pregnancy and childbirth	Poole et al (2017b)^[16]^	High consumption of coffee versus low/no consumption is associated with low birth weight and pregnancy loss.
Fracture in women	Poole et al (2017b)^[16]^	There was also an association between coffee drinking and risk of fracture in women but not in men.
Blood pressure (BP)	O’Keefe et al (2018)^[2]^	Coffee can increase in blood pressure acutely and modestly (BP) among caffeine-naive individuals but effects on habitual coffee drinkers is insignificant.
Neoplasms	Poole et al (2017b)^[16]^	High consumption of coffee associated with urinary tract and lung cancer, and acute leukemia in childhood.
Stroke	Jee et al (2020)^[17]^	The risk of ischemic stroke onset doubles transiently in an hour following coffee consumption among infrequent drinkers (less than a cup per day).
Depression	Jee et al (2020)^[17]^	Excessive caffeine intake can exacerbate depression.

In recent years, epidemiological data has indicated a global increase in insomnia and sleep-related issues. The Centers for Disease Control and Prevention in the United States have recognized sleep insufficiency as a significant public health issue, with an estimated 50 to 70 million U.S. adults facing sleep-related challenges.^[21]^ In Canada, the prevalence of sleep problems varies widely, ranging from 10% to 30% of the population.^[22]^

Insomnia, beyond the disruption of daytime sleepiness and its effect on productivity^[23]^ appears to have far-reaching effects on overall health and wellbeing. A systematic review and meta-analysis of 153 studies reported that short sleep compared to normal sleep is linked to a statistically significant increase in all-cause mortality, diabetes, hypertension, and coronary and cardiovascular diseases.^[23]^

In the Middle East, despite sleep specialists noting the increasing number of individuals grappling with sleep disorders, particularly insomnia, there is a scarcity of studies addressing its prevalence, risk factors, and impact on quality of life. Insomnia has been identified as a significant public health issue.^[24]^ A study conducted among Lebanese university students revealed that 37.1% of participants experienced poor sleep quality, primarily attributed to insomnia. This study also highlighted a significant association between anxiety and insomnia.^[24]^

In the Kingdom of Saudi Arabia, coffee is deeply ingrained in daily life, serving as both a cultural cornerstone and a symbol of hospitality.^[9]^ The aromatic beans are cultivated in the mountainous region of Jazan, the focus of our study. Recently, Saudi Arabia’s commitment to its coffee heritage was further solidified by joining the International Coffee Agreement, positioning the Kingdom to potentially become one of the world’s top producers. The significance of this was highlighted when 2022 was declared “The Year of Saudi Coffee”.^[10]^

While some studies have identified comparable patterns of caffeine consumption among students in the Gulf and the Middle East, the existing literature primarily focuses on university students, with no comprehensive population-level studies conducted, and there is limited research in Jazan, Saudi Arabia has addressed this issue. Consequently, our study aimed to examine the awareness of the prevalence of coffee consumption, its patterns, and the knowledge of its relation to insomnia among the adult population in Jazan, Saudi Arabia. The existing gap in knowledge of the prevalence of coffee consumption and the factors that influence it, especially in the Middle East and Gulf regions, and lack of population-based studies in Jazan has prompted its exploration in this research project.

Globally, the science to tackle sleep disorders and the resources allocated for targeting them are insufficient and more efforts across multiple disciplines is necessary.^[21]^

## 2. Methods

### 2.1. Study design and population

A cross-sectional, quantitative descriptive research design is applied and the study was conducted from January to June 2023 among adults aged 18 to 60 years in Jazan Province of Saudi Arabia. Subjects on medication were excluded from the study.

### 2.2. Sample size

The required sample size calculated with the formula.


$$\begin{array}{l} n=\frac{{Z_{\propto /2}}^{2}\;X\;P\;X\;\left(1-P\right)}{d^{2}} \end{array}$$

where n is the sample size, ${Z_{\propto /2}}^{2}$ is the confidence level at the 5% significance level, P is the anticipated population proportion, and d is the absolute precision. The anticipated population proportion (P) of the sample was assumed to be 50% because the maximum variance of the binomial distribution at *P* = 50%. With a 5% allowable margin of error and 95% confidence level (${Z_{\propto /2}}^{2}=4$), the sample size was calculated as 400, with a 5% anticipated non-response rate, and the desired sample size was 420. In total, 431 participants completed the survey.

### 2.3. Study instrument

A self-developed and pre-tested questionnaire was used in this study. Reliability and internal consistency of the questionnaire were also tested. The literature on coffee consumption and insomnia was studied to find out what had been covered in data collection tools and what still needed to be improved in order to create a comprehensive questionnaire. Two research yielded similar scale items.^[11,12]^ A pilot study was conducted among 30 students in 2 times with gap of week time to assess the reliability of the questionnaire. The questionnaire covers 3 domains. The first domain contains questions on demographic and health information of the participants: age, gender, marital status, education level, weight (kg), height (cm), smoking, sport activity, and medical history. The second domain for the participants’ attitudes and practices regarding coffee consumption and the last domain contained questions about the participants’ awareness of insomnia caused by coffee consumption. An online structured questionnaire was administered to all study participants.

### 2.4. Reliability testing of research instrument

The reliability of the questionnaire was tested using 2 methods including test–retest reliability and internal consistency. Test–retest intra correlation coefficients of all questions of the questionnaire were obtained as more than 0.91 and conforms the excellent consistency among the questions of the questionnaire. Cronbach alpha coefficients >0.7 was set as the most satisfactory reliability for the set of items.

### 2.5. Data collection

The final version of the survey was distributed through an online link via various social media channels to participants who met the eligibility criteria and were willing to participate. The research team managed the distribution of questionnaires.

### 2.6. Data analysis

Data were analyzed using the Statistical Package for Social Sciences version 24 (IBM, Chicago, IL) for Windows. Qualitative variables were presented as numbers and frequencies. The chi-square test was used to test the associations with background characteristics. Statistical significance was set at *P* < .05.

### 2.7. Ethical considerations

The study received ethical approval from the Standing Committee for Scientific Research at Jazan University (REC-45/08/988). Participants were assured of confidentiality and anonymity. Participants were informed of the anticipated time required for completion, study objectives, and their right to withdraw at any stage without consequences at the beginning of the questionnaire, and their consent was sought as a condition to complete the questionnaire.

## 3. Results

### 3.1. General background characteristics of the study population

Of the 431 participants who responded at the designated time, the majority were male (70.5%), <25 years of age (72.4%), single (80.7%), students (61%), nonsmokers (82.4%), engaged in some sort of sport activity (68.7%), while obese/overweight were less than a third (Table 2). While there is a higher proportion of males among the study population, the absolute number of females still allows for meaningful statistical analysis.

**Table 2:** Background characteristics of study participants.

Variable	Categories	Frequency n = 431	Percentage (%)
Gender	Male	304	304 (70.5)
	Female	127	127 (29.5)
Age	<25	312	312 (72.4)
	≥25	119	119 (27.6)
Marital status	Married	83	83 (19.3)
	Single	348	348 (80.7)
Education level	High school	115	115 (26.7)
	Bachelor or more	316	316 (73.3)
Occupation	Student	263	236 (61.0)
	Employed	105	105 (24.4)
	Unemployed/retired	63	63 (14.6)
Smoking	Yes	76	76 (17.6)
	No	355	355 (82.4)
Sports activity	Yes	296	296 (68.7)
	No	135	135 (31.3)
BMI	Under weight (≤18.49)	79	79 (18.3)
	Normal weight (18.5–24.99)	226	1126 (52.4)
	Over weight/obesity (≥25)	126	126 (29.2)

BMI = Body Mass Index.

### 3.2. Association between background characteristics and coffee consumption

Some variables of the participants’ characteristics showed a statistically significant difference in coffee consumption (Table 3). Females, married consumed more coffee than their counterparts (91.3%, *P* < .01; 92.8%, *P* < .05; respectively), while students consumed coffee (85.2%) less than both the employed and unemployed (*P* < .05). The proportion of consumption increased from underweight (79.7%) to normal weight (88.5%), and as high as 91.3% among overweight/obese (*P* < .05). Thus Table 3 captures the most important associations of coffee consumption, showing both that are statistically significantly influence more consumption (females and married), less consumption (students), as well as a dose-related increase in consumption with increased weight.

**Table 3:** Association of background characteristics with coffee consumption.

Variable	Categories	Drink coffee			*P*-value*‡*
		Yes (%)	No (%)	Total (%)	
		377 (87.5%)	54 (12.5%)	431 (100%)	
Gender	Male	261 (85.9%)	43 (14.1%)	304 (70.5%)	.003**
	Female	116 (91.3%)	11 (8.7%)	127 (29.5%)	
Age (years)	<25	271 (86.9%)	41 (13.1%)	312 (72.4%)	.388
	≥25	106 (89.1%)	13 (10.9%)	119 (27.6%)	
Marital status	Married	77 (92.8%)	6 (7.2%)	83 (19.3%)	.026*
	Single	300 (86.2%)	48 (13.8%)	348 (80.7%)	
Education	High school	96 (83.5%)	19 (16.5%)	115 (26.7%)	.107
	Bachelor or more	281 (89.0%)	35 (11.1%)	316 (73.3%)	
Occupation	Student	224 (85.2%)	39 (14.8%)	263 (61.0%)	.017*
	Employed	95 (90.5%)	4 (3.8%)	105 (24.4%)	
	Unemployed/retired	58 (92.0%)	11 (17.5%)	63 (14.6%)	
Sports activity	Yes	260 (87.8%)	36 (12.2%)	296 (68.7%)	.900
	No	117 (86.7%)	18 (13.3%)	135 (31.3%)	
BMI	Under weight (≤18.49)	63 (79.7%)	16 (20.3%)	79 (18.3%)	.044*
	Normal weight (18.5–24.99)	200 (88.5%)	26 (11.5%)	226 (52.4%)	
	Overweight/obesity (≥25)	115 (91.3%)	11 (8.7%)	126 (29.2%)	

BMI = Body Mass Index. ^‡^Chi-square test.

* Significance.

** Highly significance.

### 3.3. Coffee consumption and awareness on insomnia

**Figure 1.**
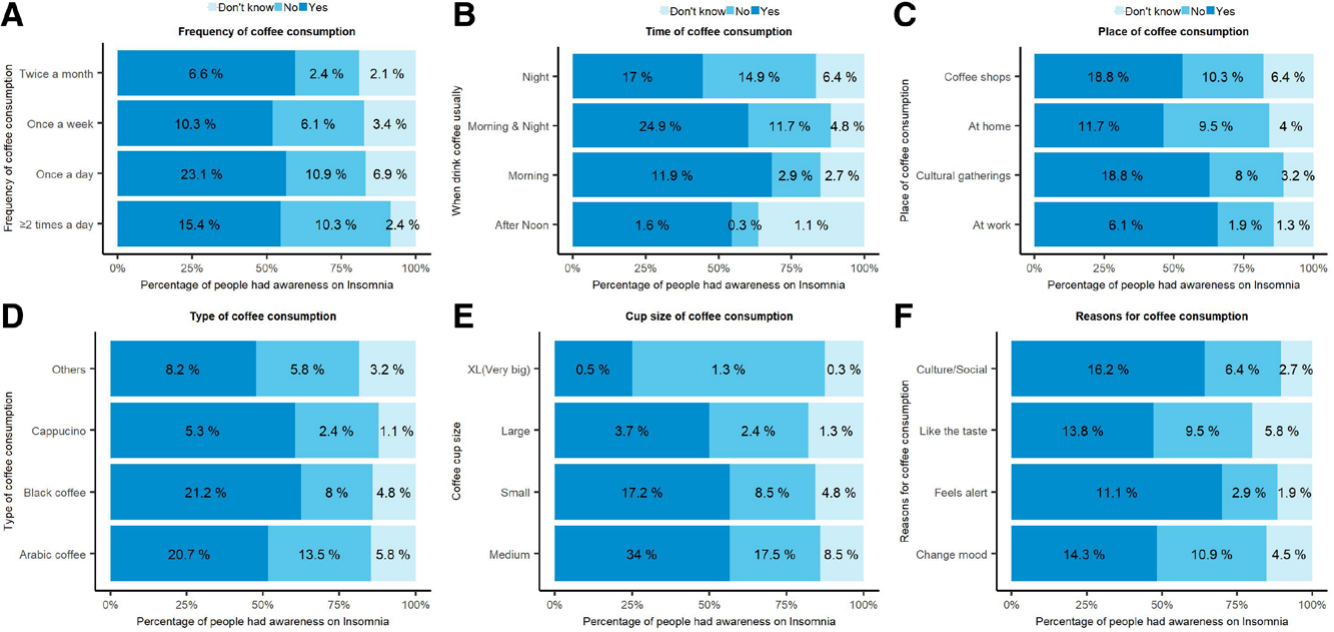
Coffee consumption and awareness on insomnia.

Regarding the coffee consumption and awareness on insomnia, the highest number of participants (40.9%) consume coffee “once a day” followed by 28.1% of the participants consuming coffee “≥2 times a day” of which 23.1% and only 15.4% were aware about the effect of coffee consumption on insomnia, respectively (Fig. 1a). Majority of participants (41.4%) consumed coffee “morning and night” of which 24.9% said coffee causes insomnia, 11.7% said no effect, and 4.8% do not know about the effect of coffee consumption on insomnia (Fig. 1b). A higher proportion (35.5%) of participants consumed coffee at coffee shops, followed by 30% at “cultural gatherings,” and 9.3% at the place (Fig. 1c). The most common type of coffee was “Arabic coffee” (40%) followed by “black coffee” (34%), of which 20.7% and 21.2% were aware of the effect of coffee consumption on insomnia, respectively (Fig. 1d). Two-thirds of the participants (60%) consumed coffee with “medium” cup size followed by one-third (30.5%) “small,” and only 2.1% of the participants consume coffee with “XL (very big)” cup size (Fig. 1e). The most common reasons for coffee consumption were “change mood” (29.7%), “like the taste” (29.1%), followed by “cultural/social” (25.3%), of which 16.2% of the participants consumed coffee even though they were aware of the effect of coffee on insomnia (Fig. 1f).

### 3.4. Awareness of insomnia caused by coffee consumption

The results in Table 4 of our study showed that age, marital status, education, and Body Mass Index (BMI) were significantly associated with awareness of insomnia caused by excessive coffee consumption. Participants in the higher age group had better awareness of insomnia due to coffee consumption than younger participants, single participants had better awareness than married participants, and those who had a bachelor’s or higher degree were better than those with a high school level. The level of awareness followed the increase in BMI, whereby underweight individuals were the least aware at a level of 48.1%, followed by those with normal weight, while the highest level of awareness was among the overweight/obese category. Gender, occupation, smoking, and sports activity were not significantly associated with knowledge of insomnia due to coffee consumption.

**Table 4:** Awareness of insomnia caused by coffee consumption.

Variable	Categories	Do you think coffee cause insomnia (sleeping difficulty)				*P*-value*‡*
		Yes (%)	No (%)	I don’t know (%)	Total (%)	
		235 (54.5%)	119 (27.6%)	77 (17.9%)	431 (100%)	
Gender	Male	170 (55.2%)	82 (26.6%)	56 (18.2%)	308 (100%)	0.767
	Female	65 (52.8%)	37 (30.1%)	21 (17.1%)	123 (100%)	
Age (years)	<25	168 (53.8%)	79 (25.3%)	65 (20.8%)	312 (100%)	0.020*
	≥25	67 (56.3%)	40 (33.6%)	12 (10.1%)	119 (100%)	
Marital status	Married	45 (49.5%)	36 (39.6%)	10 (11.0%)	91 (100%)	0.008*
	Single	190 (55.9%)	83 (24.4%)	67 (19.7%)	340 (100%)	
Education	High school	46 (40%)	41 (35.7%)	28 (24.3%)	115 (100%)	0.001**
	Bachelor or more	189 (59.8%)	78 (24.7%)	49 (15.5%)	316 (100%)	
Occupation	Student	148 (56.3%)	63 (24.0%)	52 (19.8%)	263 (100%)	0.170
	Employed	52 (52.5%)	35 (35.4%)	12 (12.1%)	99 (100%)	
	Unemployed/retired	35 (50.7%)	21 (30.4%)	13 (18.8%)	69 (100%)	
Smoking	Yes	61 (51.7%)	32 (27.1%)	25 (21.2%)	118 (100%)	0.534
	No	174 (55.6%)	87 (27.8%)	52 (16.6%)	313 (100%)	
Sports activity	Yes	170 (57.4%)	75 (25.3%)	51 (17.2%)	296 (100%)	0.178
	No	65 (48.1%)	44 (32.6%)	26 (19.3%)	135 (100%)	
BMI	Under weight (≤18.49)	38 (48.1%)	18 (22.8%)	23 (29.1%)	79 (100%)	0.040*
	Normal weight (18.5–24.99)	123 (54.4%)	70 (31.0%)	33 (14.6%)	226 (100%)	
	Over weight/obesity (≥25)	74 (58.7%)	31 (24.6%)	21 (16.7%)	126 (100%)	

‡ Chi-square test.

* Significance.

** Highly significance.

## 4. Discussion

This study explored the level of coffee consumption and its associated factors as well as the level of knowledge of the effect of coffee on sleep quality and quantity. The study revealed a high level of coffee consumption amounting to 87.5% of participants, most of which consume it at “morning and night” or night. Coffee consumption was positively associated with BMI, female gender, and being married, while students showed a lower level of consumption than both the employed and the unemployed. Approximately half of the participants believed that coffee can cause insomnia. Predictors of perceiving coffee as a possible cause of insomnia were age ≥25 years, educational level higher than school, being married, and being overweight/obese.

This study reported a coffee consumption level of 87.5% of the participants. This level is high; however, it is not far from the results obtained in other studies. Alfawaz et al (2020)^[12]^ reported a level of 88.2% among Saudi female students and 81.1% in the southern region, to which our participants belonged. Kharaba (2022)^[13]^ reports that 85% of those who consumed caffeinated beverages at least once per day. Al Ghali (2017)^[14]^ reported a level of 85.7% among United Arab Emirates students. Abdulrahman (2021)^[15]^ reported that only 8.6% of Saudi medical students never consume coffee. Students in the United States of America were found to heavily consume caffeinated drinks (92%).^[16]^

In our findings, being married was linked to more coffee consumption. This coincides with observations in.^[11]^

Coffee is usually consumed with sugar and a variety of other foods may be added.^[11]^ This can lead to speculation about the possible effect of sugar, not coffee, on weight gain among coffee consumers. In contrast, in our study, BMI was significantly associated with a higher consumption of coffee; moreover, the level increased as weight increased. This dose–response significant association was also detected by,^[12]^ and Alawadh et al^[11]^ found an association between Arabic coffee consumption and obesity as well as some additives consumed along with coffee. Knapik et al (2022) also reports a significant increase in caffeinated drinks in higher BMI.^[17]^ Age was not significantly associated with coffee consumption in the present study. Other studies have reported mixed results; Albar (2021)^[18]^ reported an inverse relationship; Lone et al (2023)^[9]^ found that in Alhasa, Saudi Arabia, older age groups consume more coffee, and the same results were obtained among Portuguese.^[19]^ Sports activity and educational level were the other variables that did not show a statistically significant relationship with coffee consumption. The students in this study consumed less coffee (85.2%) than both the employed and unemployed (*P* < .017).

About half of our participants (54.5%) thought that coffee could cause insomnia (sleeping difficulty). In this regard, this belief is held more by those who are aged 25 years or older, single, with an education level higher than school, and those in higher weight categories showing a statistically significant difference from their counterparts, while gender, smoking, sports, and occupation did not show a statistically significant difference.

## 5. Limitations

This study employed a cross-sectional design that precludes causal inferences. The study also carries the potential challenges of internet-based surveys such as the convenience sampling, selection bias, non-response as well as the subjective nature of self-reporting. Despite these limitations, the findings serve as a valuable starting point for understanding the prevalence of coffee consumption and awareness of its potential effects, laying the groundwork for future research and tailored interventions aimed at raising awareness of coffee use and insomnia. Future studies addressing the interplay between various potential confounding factors implicated in coffee consumption and its relation to insomnia.

## 6. Conclusions

This study revealed a notable prevalence of elevated coffee consumption among participants, with a substantial portion indulging in this habit during nighttime. The study adds to the understanding of how widespread is coffee consumption and factors associated with it, which can contribute to enabling informed decisions of the undesirable effects. Significantly higher coffee consumption was observed among females, married individuals, and those categorized as having a higher BMI, whereas students exhibited a comparatively lower rate. More than half of the participants believed that coffee consumption could lead to insomnia. This perception is notably associated with individuals aged 25 years or older, those with an education level beyond school, married participants, and those classified as overweight or obese. Considering the combination of heightened consumption levels, a substantial nocturnal coffee habit, and a relatively low awareness of coffee as potential causes of insomnia, there is a critical need for interventions that have been proven effective in raising awareness on this matter.

## Acknowledgments

The authors gratefully acknowledge the funding of the Deanship of Graduate Studies and Scientific Research, Jazan University, Saudi Arabia, through Project Number: GSSRD-24.

## Author contributions

**Conceptualization:** Rama M. Chandika, Fatima A. Elfaki.

**Data curation:** Fahed A. Aqili, Ahmed H. Al-Hadi.

**Formal analysis:** Rama M. Chandika.

**Methodology:** Rama M. Chandika

**Supervision:** Fatima A. Elfaki, Abdulrahman A. Alsayegh, Emadaldeen A. Alsayed.

**Validation:** Fatima A. Elfaki, Abdulrahman A. Alsayegh, Husameldin E. Khalafalla, Emadaldeen A. Alsayed, Hussin M. Hadi, Nasser M. Alhazmy, Fahed A. Aqili.

**Writing – original draft:** Rama M. Chandika, Fatima A. Elfaki, Abdulrahman A. Alsayegh, Husameldin E. Khalafalla, Emadaldeen A. Alsayed, Hussin M. Hadi, Nasser M. Alhazmy, Ahmed H. Al-Hadi.

**Writing – review & editing:** Rama M. Chandika, Fatima A. Elfaki, Abdulrahman A. Alsayegh, Husameldin E. Khalafalla, Emadaldeen A. Alsayed.
